# Supplementary material for: Cancer Malignancy Is Correlated with Upregulation of PCYT2-Mediated Glycerol Phosphate Modification of α-Dystroglycan
Source: Int J Mol Sci. 2022 Jun 15;23(12):6662. doi: 10.3390/ijms23126662 (PMC9223686; doi:10.3390/ijms23126662)
Supplement: Supplementary file 1 [file ijms-23-06662-s001.zip › ijms-1738286-supplementary.pdf]

Supplementary Materials

Cancer malignancy is correlated with upregulation of PCYT2-mediated glycerol phosphate modification of α-dystroglycan

Fumiko Umezawa[1,2,3], Makoto Natsume[4], Shigeki Fukusada[4], Kazuki Nakajima[5,6], Fumiya Yamasaki[7], Hiroto Kawashima[7], Chu-Wei Kuo[8], Kay-Hooi Khoo[1,8], Takaya Shimura[4], Hirokazu Yagi[1,3,*], and Koichi Kato[1,2,3,*]

1 Exploratory Research Center on Life and Living Systems (ExCELLS) , National Institutes of Natural Sciences, 5-1 Higashiyama, Myodaiji, Okazaki, Aichi 444-8787, Japan; c212803@ed.nagoya-cu.ac.jp (F.U.);

2 Institute for Molecular Science (IMS), National Institutes of Natural Sciences, 5-1 Higashiyama, Myodaiji, Okazaki, Aichi 444-8787, Japan
3 Graduate School of Pharmaceutical Sciences, Nagoya City University, 3-1 Tanabe-dori, Mizuho-ku, Nagoya 467-8603, Japan
4 Graduate School of Medical Sciences, Nagoya City University, 1 Kawasumi, Mizuho-cho, Mizuho-ku, Nagoya 467-8601, Japan; makoto04130510@gmail.com (M.N.); shigeki.f@hotmail.co.jp (S.F.); tshimura@med.nagoya-cu.ac.jp (T.S.)
5 Center for Joint Research Facilities Support, Research Promotion and Support Headquarters, Fujita Health University, Toyoake, Aichi 470-1192, Japan; knakaji@gifu-u.ac.jp
6 Institute for Glyco-core Research (iGCORE), Gifu University, 1-1 Yanagido, Gifu 501-1193, Japan
7 Graduate School of Pharmaceutical Sciences, Chiba University, 1-8-1 Inohana, Chuo-ku, Chiba-shi, Chiba 260-0856, Japan; axxa4826@chiba-u.jp (F.Y.); h-kawashima@chiba-u.jp (H.K.)
8 Institute of Biological Chemistry, Academia Sinica, 128 Sec. 2, Academia Road, Nankang, Taipei 115, Taiwan; kuocw@gate.sinica.edu.tw, kkhoo@gate.sinica.edu.tw (K.-H.K.)
* Correspondence: hyagi@phar.nagoya-cu.ac.jp (H.Y.); kkatonmr@ims.ac.jp (K.K.); Tel: +81-52-836-3448 (H.Y.); +81-564-59-5225 (K.K.)

Table S1: Pathological characteristics and DG2 staining of the tissue microarray.

Position	Sex	Age	Anatomic Site	Pathology	Grade	Stage	DG2
A01	M	65	Bladder, urinary	Normal	null	null	−
A02	F	39	Bladder, urinary	Transitional cell carcinoma	III	T2N0M0	+
A03	M	61	Bladder, urinary	Transitional cell carcinoma	III	T2N0M0	+
A04	F	34	Breast	Normal breast	null	null	+
A05	F	43	Breast	Invasive ductal carcinoma	II	T3N1M0	+
A06	F	75	Breast	Invasive ductal carcinoma	III	T3N0M0	+
A07	M	32	Stomach	Normal stomach	null	null	+
A08	M	66	Stomach	Adenocarcinoma	II	T3N0M0	+
B01	M	47	Stomach	Adenocarcinoma	III	T3N2M0	+
B02	F	43	Intestine, colon	Normal colon	null	null	−
B03	M	36	Intestine, colon	Adenocarcinoma	III	T3N1M0	+
B04	M	55	Intestine, rectum	Adenocarcinoma	I~II	T3N1M0	+
B05	M	30	Kidney	Normal kidney cortex	null	null	+
B06	F	79	Kidney	Cear cell carcinoma	null	T1N0M0	+
B07	M	72	Kidney	Cear cell carcinoma	null	T1N0M0	+
B08	M	35	Liver	Normal	null	null	+
C01	M	59	Liver	Hepatocellular carcinoma	II	T3N0M0	+
C02	M	19	Liver	Hepatocellular carcinoma	I~II	T2N0M0	+
C03	M	59	Lung	Normal	null	null	+
C04	M	58	Lung	Squamous cell carcinoma	II~III	T2N0M0	+
C05	F	40	Lung	Adenocarcinoma	II~III	T2N0M0	+
C08	M	42	Lymph node, axillary	Lymphoma, non-Hodgkin B cell lymphoma	null	null	+
D01	F	23	Ovary	Normal	null	null	−
D02	F	43	Ovary	Mucinous cystadenocarcinoma	III	T2N0M0	+
D03	F	58	Ovary	Endometrioid adenocarcinoma	III	T2N0M0	+
D04	F	58	Pancreas	Normal	null	null	+
D05	F	49	Pancreas	Adenocarcinoma	II	T3N1M1	+
D07	M	73	Prostate	Normal	null	null	+
D08	M	63	Prostate	Adenocarcinoma	III	T3N0M0	+
E01	M	60	Prostate	Adenocarcinoma	II	T2N0M0	+
E03	M	34	Penis	Squamous cell carcinoma	I	T1N0M0	+

E04	M	73	Skin	Melanoma	null	null	+
E05	F	46	Uterus	Normal	null	null	−
E06	F	41	Uterus, cervix	Squamous cell carcinoma	I~II	T1N1M0	+
E07	F	53	Uterus, endometrium	Adenocarcinoma	II~III	T1N1M0	+

Staining with DG2 was classified as negative; − and positive; +.

Figure S1

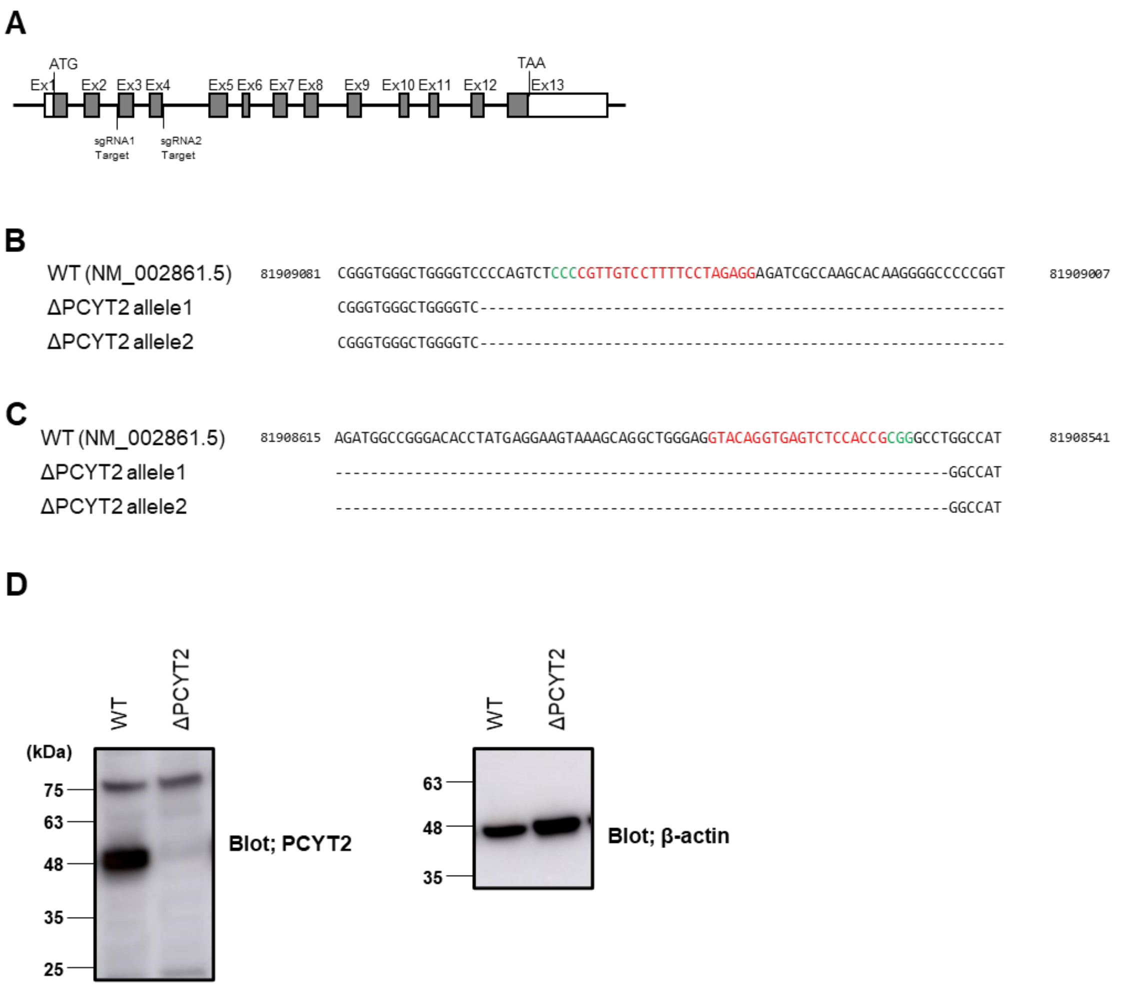

Figure S1: Editing of PCYT2 gene by the CRISPR/Cas9 system. (A) Schematic representation of the genomic target sites in the PCYT2 gene. Exons, indicated by rectangles, are numbered from Ex1 to Ex13. The coding portions of the gene are shaded. The open portions of exons 1 and 13 represent the 5′ UTR and 3′ UTR, respectively. (B, C) Genotyping results of PCYT2-KO cells. Genomic DNAs from wild-type (WT) and KO cells were isolated, and PCR amplicons flanking the CRISPR/Cas9-targeted regions were sequenced. Sequence alignments of bases (B) 81909081-81909007 and (C) 81908615-81908541 of the PCYT2 gene locations are shown. The sequences of gRNAs and PAM are labeled in red and green; -, nucleotide deletion. Numbering is based on the entire PCYT2 gene sequence using the reference gene RefSeq NM_002861.5. (D) PCYT2 protein expression level in WT and PCYT2-KO (ΔPCYT2) cells were analyzed by immunoblotting with anti-PCYT2 and anti-β-actin antibodies. The equal amount of protein in each sample was adjusted and loaded for Western blotting.

Figure S2

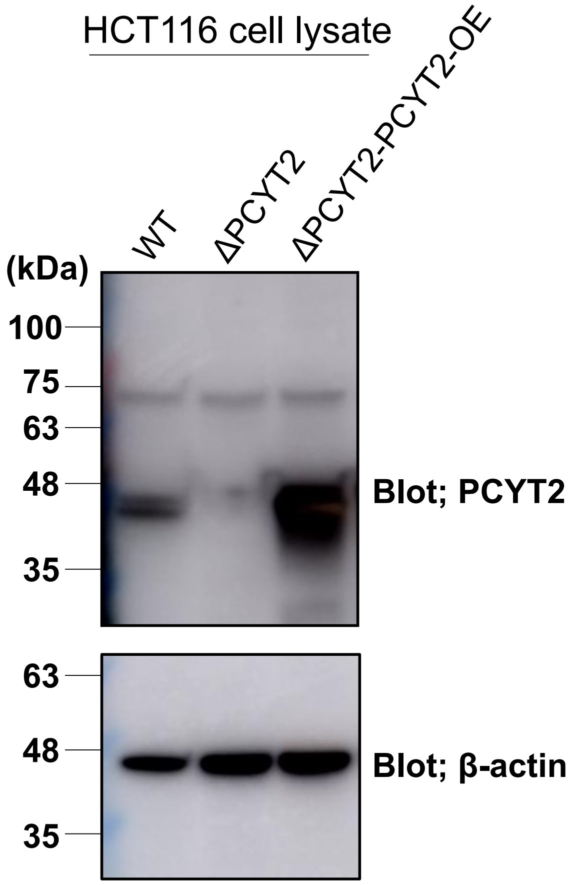

Figure S2: PCYT2 expression in HCT116 wild-type, PCYT2-KO, and PCYT2-rescue cells. Cell lysates containing equal amounts of total proteins that were prepared from HCT116 wild-type (WT), PCYT2-KO (ΔPCYT2), and PCYT2-KO rescue (ΔPCYT2-PCYT2-OE) cells were subjected to immunoblot analysis using anti-PCYT2 and anti-β-actin antibodies.
